# Bis(2-hydroxy­imino­methyl-6-methoxy­phenolato-κ^2^ *O* ^1^,*N*)nickel(II)

**DOI:** 10.1107/S1600536809005996

**Published:** 2009-02-25

**Authors:** Bing-Wen Li, Ming-Hua Zeng, Seik Weng Ng

**Affiliations:** a Department of Chemistry, Guangxi Normal University, Guilin 541000, Guangxi, People’s Republic of China; b Department of Chemistry, University of Malaya, 50603 Kuala Lumpur, Malaysia

## Abstract

The Ni atom in the title compound, [Ni(C_8_H_8_NO_3_)_2_], lies on a center of inversion in a square-planar coordination enviroment. The hydroxyl group of one anion forms a short hydrogen bond to the metal-coordinated O atom of the other anion.

## Related literature

For the structure of *o*-vanillin oxime, see: Xu *et al.* (2004 ▶). For the structure of bis­(salicylaldoximato)nickel, see: Srivastava *et al.* (1967 ▶). The title compound is expected to form complexes with nitro­gen-donor ligands as bis­(salicylaldoxinato)nickel forms such adducts; see, for example, Hultgren *et al.* (2001 ▶); Lalia-Kantouri *et al.* (1999 ▶); Ma *et al.* (2007**a* ▶,b* ▶).
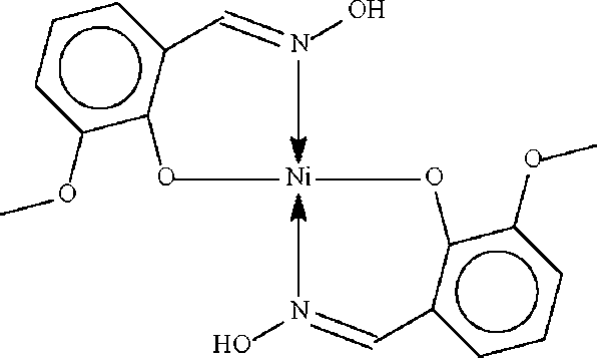


## Experimental

### 

#### Crystal data

[Ni(C_8_H_8_NO_3_)_2_]
*M* *_r_* = 391.02
Monoclinic, 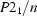

*a* = 8.3464 (8) Å
*b* = 4.8596 (4) Å
*c* = 18.735 (2) Å
β = 95.376 (2)°
*V* = 756.5 (1) Å^3^
*Z* = 2
Mo *K*α radiation
μ = 1.32 mm^−1^
*T* = 173 K
0.48 × 0.16 × 0.15 mm

#### Data collection

Bruker APEX2 diffractometer
Absorption correction: multi-scan (*SADABS*; Sheldrick, 1996 ▶) *T*_min_ = 0.570, *T*_max_ = 0.826
3461 measured reflections
1405 independent reflections
1178 reflections with *I* > 2σ(*I*)
*R*_int_ = 0.021

#### Refinement

*R*[*F* ^2^ > 2σ(*F* ^2^)] = 0.036
*wR*(*F* ^2^) = 0.105
*S* = 1.13
1405 reflections
117 parameters
H-atom parameters constrained
Δρ_max_ = 0.37 e Å^−3^
Δρ_min_ = −0.67 e Å^−3^

### 

Data collection: *APEX2* (Bruker, 2004 ▶); cell refinement: *SAINT* (Bruker, 2004 ▶); data reduction: *SAINT*; program(s) used to solve structure: *SHELXS97* (Sheldrick, 2008 ▶); program(s) used to refine structure: *SHELXL97* (Sheldrick, 2008 ▶); molecular graphics: *X-SEED* (Barbour, 2001 ▶); software used to prepare material for publication: *publCIF* (Westrip, 2009 ▶).

## Supplementary Material

Crystal structure: contains datablocks global, I. DOI: 10.1107/S1600536809005996/sj2577sup1.cif

Structure factors: contains datablocks I. DOI: 10.1107/S1600536809005996/sj2577Isup2.hkl

Additional supplementary materials:  crystallographic information; 3D view; checkCIF report

## Figures and Tables

**Table 1:** Selected bond lengths (Å)

Ni1—O1	1.827 (2)
Ni1—N1	1.866 (2)

**Table 2:** Hydrogen-bond geometry (Å, °)

*D*—H⋯*A*	*D*—H	H⋯*A*	*D*⋯*A*	*D*—H⋯*A*
O3—H3⋯O1^i^	0.84	1.86	2.492 (3)	131

Symmetry code: (i) 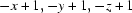.

**Fig. 1.**
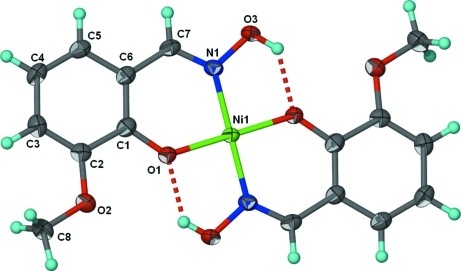
Thermal ellipsoid plot (Barbour, 2001) of Ni(C8H8NO3)2. Displacement ellipsoids are drawn at the 70% probability level, and H atoms as spheres of arbitrary radius. The dashed lines denote hydrogen bonds.

## supplementary crystallographic information

### Experimental

Nickel perchlorate hexahydrate (0.36 g, 1 mmol), 3-methoxysalicylaldoxime (0.17 g, 1 mmol) and DMF (8 ml) were placed in a 15 ml Teflon-lined autoclave. The autoclave was heated at 353 K for 3 days. The autoclave was cooled over a period of 8 h at a rate of 10 K per hour. Green crystals were collected by filtration, washed with methanol, and dried in air; yield 30% based on Ni.

### Refinement

Carbon-bound H atoms were placed at calculated positions (C–H 0.95 to 0.98 Å) and were included in the refinement in the riding model approximation, with *U*(H) set to 1.2–1.5 times *U*_eq_(C).

The crystal was original measured in the triclinic setting; the raw data when processed for absorption effects in the correct monoclinic setting had somewhat fewer reflections.

### Crystal data

**Table:**

[Ni(C_8_H_8_NO_3_)_2_]	*F*(000) = 404
*M**_r_* = 391.02	*D*_x_ = 1.717 Mg m^−^^3^
Monoclinic, *P*2_1_/*n*	Mo *K*α radiation, λ = 0.71073 Å
Hall symbol: -P 2yn	Cell parameters from 1702 reflections
*a* = 8.3464 (8) Å	θ = 2.6–26.0°
*b* = 4.8596 (4) Å	µ = 1.32 mm^−^^1^
*c* = 18.735 (2) Å	*T* = 173 K
β = 95.376 (2)°	Prism, green
*V* = 756.5 (1) Å^3^	0.48 × 0.16 × 0.15 mm
*Z* = 2	

### Data collection

**Table:**

Bruker APEX2 diffractometer	1405 independent reflections
Radiation source: fine-focus sealed tube	1178 reflections with *I* > 2σ(*I*)
graphite	*R*_int_ = 0.021
φ and ω scans	θ_max_ = 26.0°, θ_min_ = 2.2°
Absorption correction: multi-scan (*SADABS*; Sheldrick, 1996)	*h* = −8→10
*T*_min_ = 0.570, *T*_max_ = 0.826	*k* = −5→4
3461 measured reflections	*l* = −14→23

### Refinement

**Table:**

Refinement on *F*^2^	Primary atom site location: structure-invariant direct methods
Least-squares matrix: full	Secondary atom site location: difference Fourier map
*R*[*F*^2^ > 2σ(*F*^2^)] = 0.036	Hydrogen site location: inferred from neighbouring sites
*wR*(*F*^2^) = 0.105	H-atom parameters constrained
*S* = 1.13	*w* = 1/[σ^2^(*F*_o_^2^) + (0.0656*P*)^2^ + 0.2378*P*] where *P* = (*F*_o_^2^ + 2*F*_c_^2^)/3
1405 reflections	(Δ/σ)_max_ = 0.001
117 parameters	Δρ_max_ = 0.37 e Å^−^^3^
0 restraints	Δρ_min_ = −0.66 e Å^−^^3^

### Fractional atomic coordinates and isotropic or equivalent isotropic displacement parameters (Å^2^)

**Table:**

	*x*	*y*	*z*	*U*_iso_*/*U*_eq_	
Ni1	0.5000	0.5000	0.5000	0.0169 (2)	
O1	0.5102 (2)	0.2294 (4)	0.43290 (9)	0.0214 (4)	
O2	0.5912 (2)	−0.1173 (5)	0.33768 (9)	0.0262 (5)	
O3	0.2047 (2)	0.6111 (5)	0.55486 (10)	0.0257 (5)	
H3	0.2703	0.7276	0.5735	0.039*	
N1	0.2806 (3)	0.4473 (5)	0.50729 (11)	0.0192 (5)	
C1	0.3919 (3)	0.0737 (6)	0.40274 (13)	0.0210 (6)	
C2	0.4326 (3)	−0.1201 (6)	0.35041 (12)	0.0207 (6)	
C3	0.3162 (3)	−0.2898 (6)	0.31716 (13)	0.0247 (6)	
H3A	0.3443	−0.4196	0.2825	0.030*	
C4	0.1567 (3)	−0.2720 (6)	0.33415 (13)	0.0249 (6)	
H4	0.0773	−0.3896	0.3109	0.030*	
C5	0.1144 (3)	−0.0863 (6)	0.38401 (13)	0.0227 (6)	
H5	0.0057	−0.0748	0.3949	0.027*	
C6	0.2328 (3)	0.0897 (6)	0.41955 (13)	0.0201 (6)	
C7	0.1836 (3)	0.2781 (6)	0.47199 (12)	0.0212 (6)	
H7	0.0737	0.2788	0.4814	0.025*	
C8	0.6428 (4)	−0.3281 (6)	0.29197 (14)	0.0280 (7)	
H8A	0.7599	−0.3188	0.2912	0.042*	
H8B	0.5910	−0.3019	0.2433	0.042*	
H8C	0.6128	−0.5084	0.3100	0.042*	

### Atomic displacement parameters (Å^2^)

**Table:**

	*U*^11^	*U*^22^	*U*^33^	*U*^12^	*U*^13^	*U*^23^
Ni1	0.0126 (3)	0.0189 (3)	0.0194 (3)	0.00116 (19)	0.00209 (17)	−0.00161 (17)
O1	0.0155 (10)	0.0228 (11)	0.0263 (8)	−0.0005 (8)	0.0032 (7)	−0.0067 (7)
O2	0.0226 (11)	0.0263 (12)	0.0304 (10)	0.0010 (9)	0.0063 (8)	−0.0080 (9)
O3	0.0148 (10)	0.0342 (13)	0.0288 (9)	0.0006 (9)	0.0055 (7)	−0.0106 (9)
N1	0.0151 (12)	0.0226 (14)	0.0201 (10)	0.0038 (9)	0.0021 (8)	−0.0004 (8)
C1	0.0201 (14)	0.0205 (15)	0.0220 (12)	−0.0015 (11)	−0.0004 (10)	0.0023 (10)
C2	0.0205 (14)	0.0220 (15)	0.0197 (11)	0.0016 (12)	0.0021 (9)	0.0038 (10)
C3	0.0293 (16)	0.0220 (16)	0.0225 (11)	0.0004 (12)	0.0016 (10)	−0.0021 (11)
C4	0.0235 (15)	0.0253 (17)	0.0248 (12)	−0.0053 (12)	−0.0039 (10)	0.0023 (11)
C5	0.0186 (14)	0.0259 (16)	0.0235 (12)	−0.0039 (12)	0.0011 (10)	0.0040 (11)
C6	0.0190 (14)	0.0199 (14)	0.0210 (11)	−0.0014 (11)	−0.0001 (10)	0.0024 (10)
C7	0.0140 (13)	0.0253 (15)	0.0242 (12)	0.0014 (11)	0.0006 (9)	0.0019 (11)
C8	0.0287 (16)	0.0283 (18)	0.0278 (13)	0.0033 (12)	0.0065 (11)	−0.0041 (11)

### Geometric parameters (Å, °)

**Table:**

Ni1—O1	1.827 (2)	C2—C3	1.378 (4)
Ni1—O1^i^	1.827 (2)	C3—C4	1.400 (4)
Ni1—N1	1.866 (2)	C3—H3A	0.9500
Ni1—N1^i^	1.866 (2)	C4—C5	1.369 (4)
O1—C1	1.328 (3)	C4—H4	0.9500
O2—C2	1.367 (3)	C5—C6	1.424 (4)
O2—C8	1.427 (3)	C5—H5	0.9500
O3—N1	1.391 (3)	C6—C7	1.431 (4)
O3—H3	0.8400	C7—H7	0.9500
N1—C7	1.292 (3)	C8—H8A	0.9800
C1—C6	1.395 (4)	C8—H8B	0.9800
C1—C2	1.423 (4)	C8—H8C	0.9800
			
O1—Ni1—O1^i^	180.00 (7)	C4—C3—H3A	119.8
O1—Ni1—N1	93.50 (9)	C5—C4—C3	120.5 (3)
O1^i^—Ni1—N1	86.50 (9)	C5—C4—H4	119.7
O1—Ni1—N1^i^	86.50 (9)	C3—C4—H4	119.7
O1^i^—Ni1—N1^i^	93.50 (9)	C4—C5—C6	120.2 (3)
N1—Ni1—N1^i^	180.00 (12)	C4—C5—H5	119.9
C1—O1—Ni1	128.47 (17)	C6—C5—H5	119.9
C2—O2—C8	116.7 (2)	C1—C6—C5	119.7 (3)
N1—O3—H3	109.5	C1—C6—C7	122.1 (3)
C7—N1—O3	113.0 (2)	C5—C6—C7	118.3 (2)
C7—N1—Ni1	128.46 (19)	N1—C7—C6	123.5 (2)
O3—N1—Ni1	118.51 (16)	N1—C7—H7	118.2
O1—C1—C6	123.9 (2)	C6—C7—H7	118.2
O1—C1—C2	117.0 (2)	O2—C8—H8A	109.5
C6—C1—C2	119.1 (3)	O2—C8—H8B	109.5
O2—C2—C3	125.5 (2)	H8A—C8—H8B	109.5
O2—C2—C1	114.3 (2)	O2—C8—H8C	109.5
C3—C2—C1	120.2 (2)	H8A—C8—H8C	109.5
C2—C3—C4	120.4 (3)	H8B—C8—H8C	109.5
C2—C3—H3A	119.8		
			
N1—Ni1—O1—C1	2.2 (2)	O2—C2—C3—C4	−179.3 (2)
N1^i^—Ni1—O1—C1	−177.8 (2)	C1—C2—C3—C4	0.4 (4)
O1—Ni1—N1—C7	−2.1 (2)	C2—C3—C4—C5	−0.1 (4)
O1^i^—Ni1—N1—C7	177.9 (2)	C3—C4—C5—C6	−0.4 (4)
O1—Ni1—N1—O3	179.66 (18)	O1—C1—C6—C5	179.9 (2)
O1^i^—Ni1—N1—O3	−0.34 (18)	C2—C1—C6—C5	−0.3 (4)
Ni1—O1—C1—C6	−1.4 (4)	O1—C1—C6—C7	−0.3 (4)
Ni1—O1—C1—C2	178.79 (17)	C2—C1—C6—C7	179.5 (2)
C8—O2—C2—C3	−7.2 (4)	C4—C5—C6—C1	0.6 (4)
C8—O2—C2—C1	173.0 (2)	C4—C5—C6—C7	−179.2 (2)
O1—C1—C2—O2	−0.6 (3)	O3—N1—C7—C6	179.5 (2)
C6—C1—C2—O2	179.6 (2)	Ni1—N1—C7—C6	1.3 (4)
O1—C1—C2—C3	179.6 (2)	C1—C6—C7—N1	0.4 (4)
C6—C1—C2—C3	−0.2 (4)	C5—C6—C7—N1	−179.8 (3)

Symmetry codes: (i) −*x*+1, −*y*+1, −*z*+1.

### Hydrogen-bond geometry (Å, °)

**Table:**

*D*—H···*A*	*D*—H	H···*A*	*D*···*A*	*D*—H···*A*
O3—H3···O1^i^	0.84	1.86	2.492 (3)	131

Symmetry codes: (i) −*x*+1, −*y*+1, −*z*+1.
